# Chaihu-Shugan-San Administration Ameliorates Perimenopausal Anxiety and Depression in Rats

**DOI:** 10.1371/journal.pone.0072428

**Published:** 2013-08-27

**Authors:** Shujiao Chen, Tetsuya Asakawa, Shanshan Ding, Linghong Liao, Lingyuan Zhang, Jianying Shen, Jie Yu, Kenji Sugiyama, Hiroki Namba, Candong Li

**Affiliations:** 1 Research base of TCM syndrome, Fujian University of Traditional Chinese Medicine, Minhou Fuzhou City, P.R of China; 2 Department of Neurosurgery, Hamamatsu University School of Medicine, Hamamatsu-city, Japan; University of Insubria, Italy

## Abstract

Chaihu-Shugan-San (CSS) is a traditional Chinese herbal formula that is widely used for treating perimenopausal symptoms in China; however, its mechanisms remain unknown. The present study was designed to investigate potential CSS mechanisms in rats with unpredicted chronic mild stress (UCMS) and normally aging rats (52 weeks of age). We performed the sucrose consumption test along with the forced swimming test to confirm depression-like behavior and the open field test (OFT) to confirm anxiety-like behavior in the animals. In addition, we used an enzyme-linked immunosorbent assay to measure serum and hippocampal estradiol (E_2_) levels and a quantitative real-time polymerase chain reaction to assess hippocampal mRNA levels of estrogen receptors (ERs) α and β as well as G protein-coupled receptor 30 (GPR30). We found that CSS administration resulted in a significant increase in the ratio of hippocampal ERα and ERβ mRNA (ERα/ERβ ratio) in UCMS rats (*p*<0.001). However, no significant changes were observed in E_2_ levels, ERα mRNA expression, and GPR30 mRNA expression. In contrast, changes in ERα/ERβ mRNA ratio were sensitively associated with changes in mood states in the animal models. These findings suggest that enhancement of ERα/ERβ ratio may play a role in the pharmacological mechanisms of CSS. Furthermore, this ratio can be employed as a potential index for evaluating mood states in animal models and can be considered as a therapeutic target for perimenopausal anxiety and depression in the future.

## Introduction

Perimenopause is a period of profound transition in women’s lives. In addition to physical symptoms such as hot flashes and night sweats, psychological changes such as anxiety, depression, and memory loss are major complaints among perimenopausal women [1], [2], [3]. However, the pathomechanisms underlying these psychological symptoms remain unclear. It is believed that serum estrogen levels progressively decrease during perimenopause, and this may cause neurological problems. Gyllstrom (2007) reported that depressive symptoms are accompanied by a significant decrease in plasma follicle-stimulating hormone (FSH) levels [4]. Moreover, Freeman (2004) reported that depressive symptoms can follow fluctuations in FSH levels [5]. In addition, it has been reported that women with a history of psychological problems are at increased risk for an earlier perimenopausal transition with higher FSH and luteinizing hormone (LH) levels and lower estradiol (E_2_) levels [6]. Other studies have elucidated the relationship between estrogen receptor (ER) levels and perimenopausal stress. Takeo (2005) evaluated the association of cytosine–adenine repeat polymorphism of the ERβ gene with menopausal symptoms and reported four variants of the cytosine adenine repeat polymorphism [7]. Walf (2007) directly administered ERβ-selective ligands to the hippocampus and relieved the symptoms of depression and anxiety; this suggests a potential role of hippocampal ERβ in the development of neurological symptoms [8].

Chaihu-Shugan-San (CSS) is a popular traditional Chinese herbal medicine that is recorded in a Chinese medical classic “Jingyue Quanshu.” It consists of seven Chinese herbs: *Bupleurum chinense* DC (chai-hu), *Pericarpium citri reticulatae* (chen-pi), *Radix paeoniae alba* (bai-shao), *Fructus aurantii* (zhi-qiao), *Rhizoma cyperi* (xiang-fu), *Ligusticum chuanxiong* Hort (chuan-qiong), and *Glycyrrhiza uralensis* Fisch (gan-cao). Chemically, the constituents of CSS are complex and include monoterpene glycosides, galloyl glucoses, phenolic compounds, lactones, flavonoids, and triterpene saponins [9], [10], [11], [12], [13]. In traditional Chinese medicine (TCM), CSS is used to treat a wide variety of symptoms caused by liver qi stagnation, including perimenopausal anxiety and depression [14], [15], [16]. For a long time, CSS has been used as an alternative therapy for treating anxiety and depression [14], [15], [16] and perimenopausal psychological symptoms [17].

However, the mechanisms by which CSS alleviates perimenopausal psychological problems remain unknown, and this has limited its wider use. In the present study, we investigated the efficacy of CSS in treating depression-like behavior and anxiety-like behavior in animal models. Furthermore, we examined the potential mechanisms of action of CSS by measuring serum and hippocampal E_2_ levels and hippocampal mRNA expression of ERα, ERβ, and G protein-coupled receptor 30 (GPR30) after CSS administration.

## Materials and Methods

### Ethics Statement

All rats were treated as per the National Institute of Health Guidelines for the Care and Use of Laboratory Animals. All experiments were approved by the Animal Care and Use Committee of the Fujian University of Traditional Chinese Medicine [permission number: SYXK (Min) 2009-0001]. All surgical procedures were performed under sodium pentobarbital anesthesia, and all efforts were made to minimize suffering.

### Preparation of CSS

The standard CSS solution was prepared on the basis of the formula provided in the Chinese Pharmacopoeia (CP; Version 2010) (Table 1). Raw components of the CSS formulation were mixed and crushed into small pieces and were soaked in distilled water for 30 min. Ten volumes of cold deionized water was added to the herbs and boiled for 30 min; thereafter, the marc was extracted eight times in the same volume of water. The aqueous extract was collected and combined with the former extract, filtered with filter paper, and condensed to 1/10 the volume by rotary evaporation at 80±5 rpm and 60°C ±1°C.

**Table 1:** The ingredients of the Chaihu-Shugan-San (CSS) solution.

Chinese name	Ping Yin	English name	Part used	Proportion of ingredients (100%)
白芍	bai-shao	*Radix paeoniae alba*	Dried root	14.29
陈皮	chen-pi	*Pericarpium citri reticulatae*	Dried tangerine peel	19.05
枳壳	zhi-qiao	*Fructus aurantii*	Dried orange peel	14.29
香附	xiang-fu	*Rhizoma cyperi*	Dried root	14.29
川芎	chuan-qiong	*Ligusticum chuanxiong* Hort.	Dried root	14.29
甘草	gan-cao	*Glycyrrhiza uralensis* Fisch.	Dried root	4.74
柴胡	chai-hu	*Bupleurum chinense DC*	Dried root	19.05

### Animals and Administration

Sixty female Sprague–Dawley rats (SPF, Beijing Vital River Laboratory Animal Technology Co, Beijing, China) were housed in humidity- (55%) and temperature-controlled (23°C) rooms with a 12-h light/dark cycle and food and water supply.

The unpredictable chronic mild stress (UCMS) model was generated using established methods as described previously [18]. Briefly, rats were exposed to the following nine stressors: damp sawdust (12 h), nip tail (1 min), food deprivation (12 h), water deprivation (12 h), forced swimming in cold water (5°C, 2 min), forced swimming in hot water (45°C, 2 min), cage tilt (45°, 12 h), exposure to an empty bottle (12 h), and overnight illumination. The rats randomly received two of these stressors per day, and the same stressor was never applied for 2 consecutive days. The entire stress procedure lasted for 3 weeks.

Twelve-week-old female rats (250–280 g, *n* = 12) were used as normal young controls (CON). A total of 24 normally aging rats (age, 52 weeks; weight, 370–380 g) that were age- and weight-matched with the UCMS rats were randomly assigned into two control groups: a naturally aging control group (ACON without CSS, *n* = 12) and a normally aging control group (ACON with CSS, *n* = 12). We also created two UCMS rat groups, namely UCMS rats not administered CSS (UCMS without CSS, *n* = 12) and UCMS rats administered CSS (UCMS with CSS, *n* = 12).

Rats belonging to the ACON with CSS and UCMS with CSS groups were administered CSS orally by gavage [1000 mg/kg/body weight (BW)], whereas those belonging to the CON, ACON without CSS, and UCMS without CSS groups were given water (10 ml/kg BW) by gavage. CSS was administered simultaneously when the UCMS model was being established. CSS or water was administered 1 h before each test to relieve the potential stress induced by gavage. Administration was continuous during the entire period of behavioral tests.

The stages of the estrous cycle were monitored by microscopic examination of vaginal smears. We made sure all rats in a study were on the diestrus stage of the cycle.

### Behavioral Assessments

**Figure 1:**
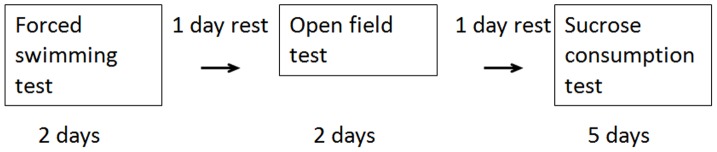
Protocol for the behavioral tests. The interval between the blocks was 1 day.

We selected the sucrose consumption test and forced swimming test (FST) for evaluating depression-like behavior and the open field test (OFT) for evaluating anxiety-like behavior. The protocol of the behavioral tests is shown in Figure 1. The interval between each test was 1 day (Figure 1.).

### Sucrose Consumption Test

The sucrose-consumption test is often used to measure depression-like behavior in rats by the evaluation of the hedonic state or the ability to gain pleasure [19]. Impairment during the test is used to imitate clinical depression [20]. The test was performed as described in previous studies [19], [21]. In brief, rats were placed in a test cage, which was similar to the home cage, and sucrose intake (consumption of 20% sucrose solution in 15 min) was measured by comparing the weight of bottles before and after the test. The entire procedure was performed in 5 consecutive days. We only used the data for sucrose intake on the 5th day as the index of sucrose intake for statistical evaluation.

### FST

FST is another classical behavioral test for measuring depression-like behavior in rats [22]. The index of the test is immobility time (IT). We followed a modified protocol described by Sarkisova [19], [21]. Briefly, the test was performed in a plastic cylinder (internal diameter, 38 cm; height, 47 cm). The height and temperature of the water were 38 cm and 25±1°C, respectively. We omitted the initial 15-min habituation session as described in the previous study [19], and rats were individually placed in the tank and forced to swim for 5 min. The total duration of immobilization (including passive swimming) was recorded. The criterion for immobilization or passive swimming was vertical floating in the water and the exhibition of only those movements that were necessary to keep the head above the water. The entire procedure was performed in 2 consecutive days from 10∶00 am to 12∶00 am. The first day was training day while the second day was test day. We maintained the same time duration for the training day and the actual test day. After the test, animals were removed and cared for in their home cage.

### OFT

OFT was performed to assess emotional behavior and locomotor activity according to previously described methods [19], [23]. Briefly, we used an open-field device comprising a four-sided 80 × 80 × 40-cm wooden enclosure with black walls and a white floor that was divided into 25 equal squares by black lines. Tests were performed in a darkened, quiet room lit by two 40-W light bulbs suspended above the center of the open field (light level, 365 lux). Each rat was gently placed onto the middle square of the open-field apparatus in a direction facing away from the observer and allowed to move freely for 5 min. Animal movements in the field during the test time were quantified by counting the number of squares crossed and that of rearings. The number of entries into the central area was also counted in addition to the time spent in the central area.

### Tissue Preparation

After the behavioral tests, all the rats were deeply anesthetized with intraperitoneal injections of sodium pentobarbital (50 mg/kg BW). The hippocampus was removed, immediately frozen in liquid nitrogen, and stored at −70°C. Frozen hippocampus specimens were homogenized in 0.2-N HClO_4_ and centrifuged at 10,000 *g* for 5 min at 4°C. The supernatant was collected and filtered through a 0.2-µm filter for measuring E_2_ levels. The precipitate was used for mRNA detection.

### Measurement of Serum and Hippocampal E_2_ Levels

The pH of the supernatant was neutralized to 7–8 with one volume of 1-M borate buffer (pH, 9.25), and this was followed by centrifugation at 10,000 *g* for 1 min at 4°C. E_2_ levels in 100-µl aliquots of the samples were analyzed using an enzyme-linked immunosorbent assay (ELISA) kit (Cayman Chemical Company, Ann Arbor, Michigan, USA) with a microplate reader (BioTekGC-2010, USA) at 405 nm. Serum E_2_ levels were also measured by the same method. The intra- and interassay coefficients of variation (CVs) were 22.2% and 14.7% and 15.8% and 6.2% in the serum and hippocampus, respectively.

### Measurement of Hippocampal mRNA Expression Using Quantitative Real-time Reverse Transcription Polymerase Chain Reaction (RT-PCR)

The residual deposits of hippocampal tissues were homogenized with 1 ml of Trizol reagent (Invitrogen, CA, USA), following which total RNA was extracted as per the manufacturers’ instructions. Homogenates were mixed in chloroform and centrifuged at 12,000 g for 15 min at 4°C. RNA was precipitated with isopropanol and centrifuged at 12,000 g for 15 min at 4°C. The precipitated RNA was treated with 75% ethanol, followed by centrifugation at 7,500 g for 5 min at 4°C. The total RNA pellet was air-dried and resuspended in RNase-free water and the RNA level was measured using the Fluostar Optima spectrometer (BMG Labtech, Ortenberg, Germany). After measuring the RNA level and the purity of the sample using the Fluostar Optima Spectrometer, 1.5 µg of total RNA was reverse transcribed using a high-capacity cDNA reverse transcription kit (Applied Biosystems, Foster City, CA, USA) as per the manufacturers’ instructions. The reverse transcription reaction was performed in a thermal cycler (TaKaRa PCR Thermal Cycler, Biological Engineering Company Limited, Tokyo, Japan) using the Applied Biosystems High Capacity cDNA system (Gene Amp PCR System 9700, Applied Biosystems, CA, USA). Quantitative real-time RT-PCR reactions were performed with 10 ng of cDNA template using SYBR Green PCR master mix (Applied Biosystems, Carlsbad, USA) and were run on the quantitative real-time RT-PCR machine (Applied Biosystems, Prism 7000). The housekeeping gene GAPDH was selected as an internal control for the analysis of GAPDH mRNA expression as previously described [24]. The thermocycling reaction was carried out at 60°C for 1 min, 95°C for 10 min, followed by 40 cycles of 95°C for 15 s and 60°C for 1 min. The primer sequences used are shown in Table 2. The relative mRNA expression of target genes was calculated using the 2-ΔΔCt method. Relative amplification efficiencies of the primers were tested and observed to be similar. CON group expression levels were designated as 100%, and the relative expression levels in the other groups were recorded as values relative to the expression levels in CON group.

**Table 2:** Primers for quantitative real-time RT-PCR (Rat).

Gene	Primer sequences	Anneal temperature (°C)	Product (bp)
GAPDH	F:5′GGAAAGCTGTGGCGTGAT3′ R:5′AAGGTGGAAGAATGGGAGTT3′	60	308
GPR30	F:5′CGCTCAAGGCAGTCATACCA3 ′ R:5′CCCCTGTCCGTTTTCCTCTA3′	60	96
ERβ	F:5′TGCTGGATGGAGGTGCTAATG3′ R:5′GAGGTCGGGAGCGAAAATG3′	60	81
ERα	F:5′GGAGACTCGCTACTGTGCTGTGT3′ R:5′AGTCATCTCTCTGACGCTTGTGC3′	60	281

### Statistical Analyses

Data were analyzed using SPSS 19.0.0 software (SPSS Inc., IL, USA). Three-way analysis of variance (ANOVA) followed by Bonferroni post-hoc correction was performed for multiple comparisons. The variables included age, treatment, and stress exposure (UCMS processes). The type I error in ANOVA was accepted as p≤0.05. Post-hoc Bonferroni correction was applied only if the p value in ANOVA was <0.05. In this case, the type I error was corrected to p≤0.005 [α/k = 0.05/10; k = 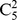 = 5 × (5 − 1)/2]. All values are presented as means ± standard error of means (SEMs). All the tests were two-sided.

## Results

### Validation of the Rat Groups used in this Study

**Figure 2:**
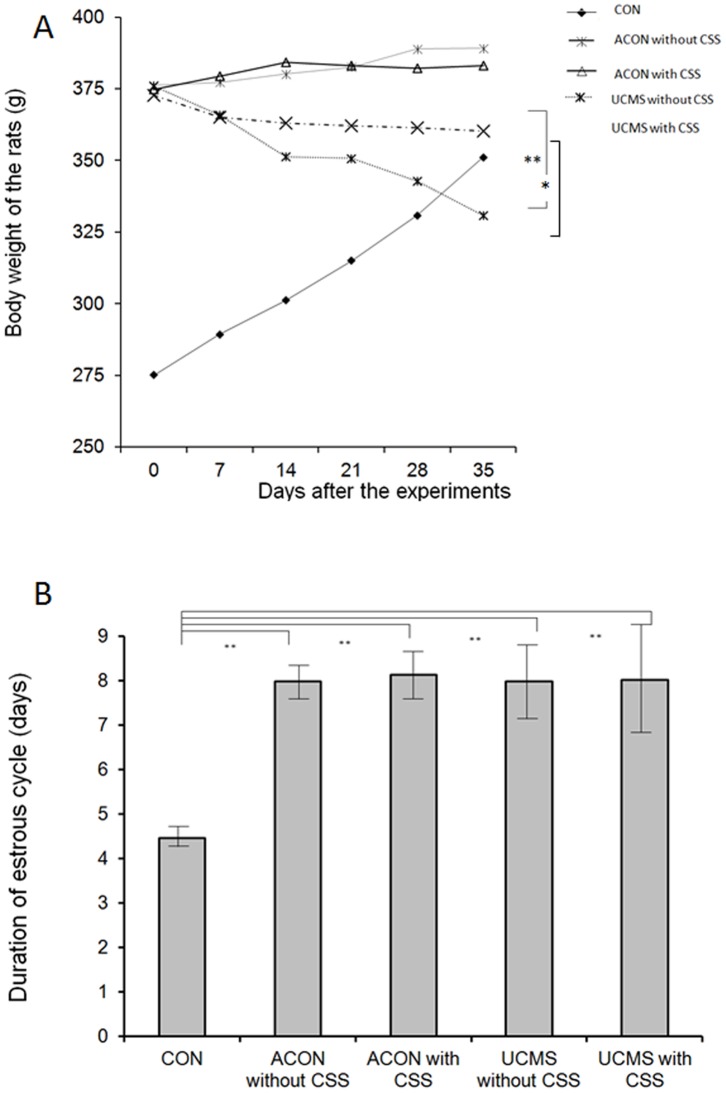
Validation of the rat groups used in this study. A: Changes in body weight (BW) of the rats during the entire experimental period. BW of the young normal control rats (CON) increased sharply with an increase in time, that of the normally aging rats without CSS administration (ACON without CSS) increased gradually; that of the normally aging rats with CSS administration (ACON with CSS) was the same as that of the normally aging rats without CSS administration (*p*>0.05), that of the UCMS rats without CSS administration (UCMS without CSS) decreased sharply, and that of the UCMS rats with CSS administration (UCMS with CSS) decreased gradually. CSS administration decreased the weight loss in the UCMS rats (*p<0.005). B: Duration of estrous cycle in the rats. Duration of estrous cycle was significantly shorter in the CON rats than in the ACON and UCMS rats, while no significant difference was found between ACON and UCMS rats and between rats who received CSS and those who did not (**p<0.001).

Figure 2A shows the dynamic changes in BW of all rats during the experimental period. BW in the UCMS without CSS group decreased sharply, while that in the UCMS with CSS group decreased gradually, indicating that CSS administration may have ameliorated the depressive/anxiety states in the UCMS rat model (UCMS with CSS vs. UCMS without CSS, *p*<0.005; Figure 2A).

Figure 2B shows the changes in the duration of estrous cycle of the rats. The duration of estrous cycle was significantly prolonged in the aging rats (ACON and UCMS) compared with that in the normal young rats (CON vs. ACON without CSS, ACON with CSS, UCMS with CSS, and UCMS without CSS, *p*<0.001; Figure 2B). We also identified a regular estrous cycle in the CON group rats and a disturbed cycle in the aging rats. Such changes in the duration of estrous cycle were not affected by the UCMS processes (*p*>0.05) and CSS administration (*p*>0.05). By monitoring the changes in the duration of estrous cycle, we confirmed that the aging rats used in this study mimicked perimenopausal women.

### CSS Affected Depression-like Behavior in the UCMS Rats

**Figure 3:**
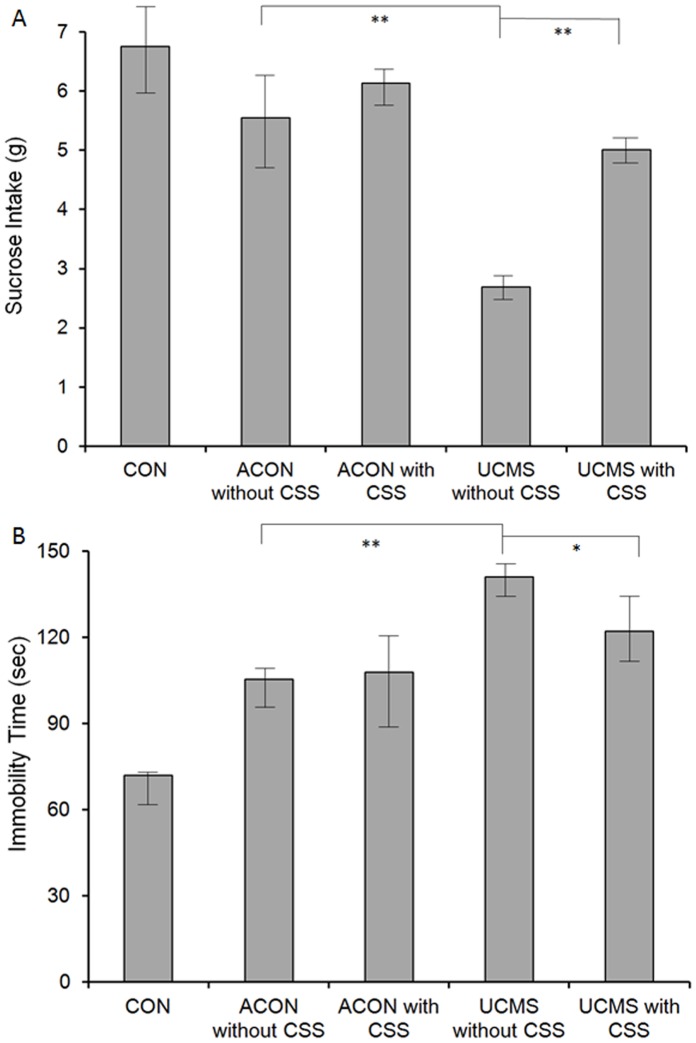
Behavioral tests for depression-like behavior in rats. A. Results of the sucrose consumption test. Sucrose intake was significantly decreased in the UCMS without CSS group compared with that in the ACON without CSS groups (*p*<0.001). On the other hand, sucrose intake in the UCMS with CSS group was significantly increased compared with that in the UCMS without CSS (*p*<0.001) group. B: Results of the forced swimming test (FST). The immobility time was significantly longer in the UCMS without CSS group than in the ACON without CSS group (*p*<0.001). However, immobility time in the UCMS with CSS group was significantly shorter than that in the UCMS without CSS group (*p*<0.005). The depressive state in the UCMS rats was verified (ACON without CSS vs. UCMS without CSS). CSS administration was effective for treating depressive symptoms in the UCMS rats (UCMS with CSS vs. UCMS without CSS). Values are means ± SEM. *p<0.005, **p<0.001. The *p*-value was established by analysis of variance (ANOVA). The post-hoc Bonferroni test was not applied when the *p*-value was >0.05 as per ANOVA).

Figure 3A shows the results of the sucrose consumption test. We found that sucrose intake was affected by the UCMS processes in the aging rats. Sucrose intake in the UCMS without CSS group was significantly lower than that in the ACON without CSS group (*p*<0.001). Moreover, sucrose intake improved by CSS administration. Sucrose intake in the UCMS with CSS group was significantly higher than that in the UCMS without CSS group (*p*<0.001). We thus found that sucrose intake was not affected by age (*p*>0.05).

Figure 3B shows the results of the FST. We found that the immobility time was affected by the UCMS processes in the aging rats, and it was significantly prolonged in the UCMS without CSS group compared with that in the ACON without CSS group (*p*<0.001). Furthermore, the immobility time was ameliorated by CSS administration. The immobility time in the UCMS with CSS group was significantly shorter than that in the UCMS without CSS group (*p*<0.005). We thus found that the immobility time was not affected by age (*p*>0.05). The abovementioned findings confirmed depression-like behavior in the UCMS rats and revealed that CSS administration ameliorated this behavior (Figure 3).

### CSS Affected Anxiety-like Behavior in the UCMS Rats

**Figure 4:**
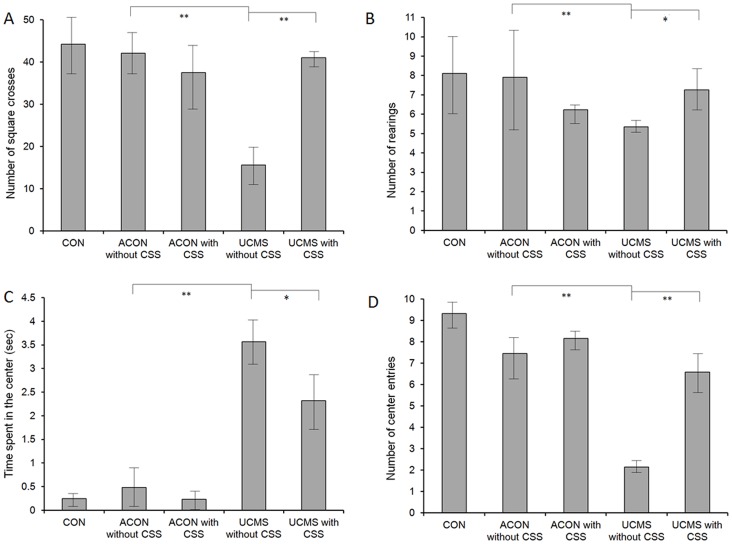
Open field test for anxiety-like behavior. OFT indices, including number of squares crossed (Figure 4A), number of rearings (Figure 4B), and number of center entries (Figure 4D) were significantly lower in the UCMS without CSS group than in the ACON without CSS group (*p*<0.001). Moreover, the index of time spent in the center (Figure 4C) was significantly longer in the UCMS without CSS group than in the ACON without CSS (*p*<0.001) group, confirming the anxiety state in the UCMS rats (ACON without CSS vs. UCMS without CSS). Furthermore, the number of square crosses (Figure 4A), number of rearings (Figure 4B), and number of center entries (Figure 4D) were significantly higher in the UCMS with CSS group than in the UCMS without CSS group. Moreover, the time spent in the center (Figure 4C) was significantly shorter in the UCMS with CSS group than in the UCMS without CSS group [numbers of square crosses (Figure 4A) and center entries (Figure 4D), p<0.001; numbers of rearings (Figure 4B) and time spent in the center (Figure 4C), p<0.005], showing that CSS administration was effective for releasing the stressful state in UCMS rats (UCMS with CSS vs. UCMS without CSS). Values are means ± SEM. *p<0.005, **p<0.001. The *p*-value was established by analysis of variance (ANOVA). The post-hoc Bonferroni test was not applied when the *p*-value was >0.05 as per ANOVA).

The OFT indices were affected by the UCMS processes. Poorer performance, including a lower number of squares crossed (Figure 4A), rearings (Figure 4B), and center entries (Figure 4D), and longer time spent in the center (Figure 4C) by the rats in the UCMS without CSS group verified anxiety-like behavior in the UCMS rats (UCMS without CSS group vs. ACON without CSS group, *p*<0.001). The OFT indices were also affected by CSS administration. After CSS administration, animals in the UCMS with CSS group achieved better performance in the OFT compared with animals in the UCMS without CSS group [numbers of square crosses (Figure 4A) and center entries (Figure 4D), p<0.001; numbers of rearings (Figure 4B) and time spent in the center (Figure 4C), p<0.005]; however, no significant difference was observed between the ACON with CSS and ACON without CSS groups. These results indicate that CSS was effective for treating anxiety-like behavior only 13in the UCMS rats (Figure 4). The OFT results were not affected by age (*p*>0.05).

### Effects of CSS Administration on Serum and Hippocampal E_2_ Levels

**Figure 5:**
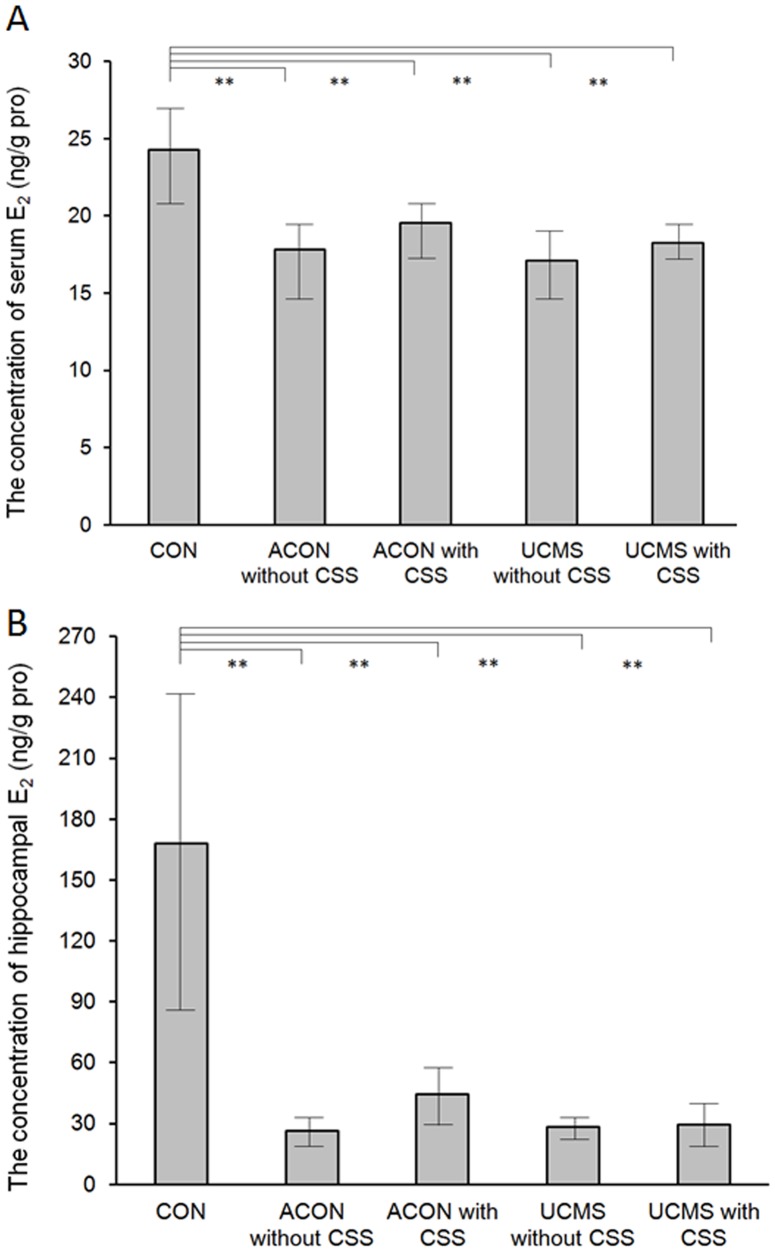
Serum and Hippocampal E_2_ levels. The results for serum (Figure 5A.) and hippocampal (Figure 5B.) E_2_ levels were analogous. The E_2_ levels in the CON group were significantly higher than those in the other groups (*p*<0.001), but no difference was observed among the other groups. Values are means ± SEM. **p<0.001. The *p*-value was established by analysis of variance (ANOVA). The post-hoc Bonferroni test was not applied when the *p*-value was >0.05 as per ANOVA).

Serum (Figure 5A) and hippocampal (Figure 5B) E_2_ levels were analogous in this study. We found that E_2_ levels were only affected by age. E_2_ levels in the CON rats were significantly higher than those in the aging rats (CON group vs. ACON without CSS, ACON with CSS, UCMS without CSS, and UCMS with CSS groups, *p*<0.001). E_2_ levels were not affected by both UCMS processes (ACON groups vs. UCMS groups, *p*>0.05) and CSS administration (ACON without CSS group vs. ACON with CSS group and UCMS without CSS group vs. UCMS with CSS group; *p*>0.05; Figure 5).

### Effects of CSS Administration on Hippocampal mRNA Expression of ERα, ERβ, and GPR30 in the Aging or UCMS Rats

**Figure 6:**
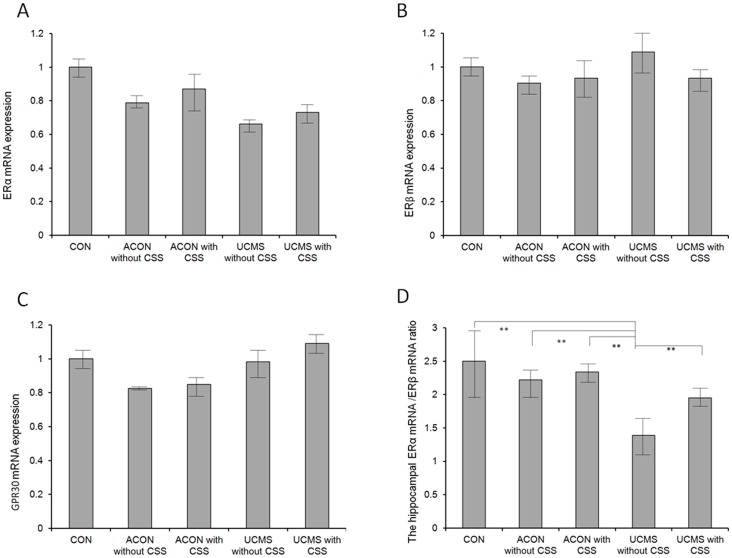
Hippocampal mRNA expression of ERα, ERβ, and GPR30 and the ERα/ERβ mRNA ratio. A: No significant difference was observed among all rats. B: ERβ mRNA expression showed no significant difference among all groups. C: GPR30 mRNA expression showed no significant difference among all groups. D: The ERα/ERβ ratio was significantly lower in the UCMS without CSS group than in the other groups (*p*<0.001). Therefore, this ratio was sensitively affected in the UCMS rats (UCMS without CSS vs. CON and ACON). The ERα/ERβ ratio was higher in the UCMS with CSS group than in the UCMS without CSS group (*p*<0.001). This ratio was significantly ameliorated by CSS administration. Values are means ± SEM. **p<0.001. The *p*-value was established by analysis of variance (ANOVA). The post-hoc Bonferroni test was not applied when the *p*-value was >0.05 as per ANOVA).

The results for ERα mRNA expression (Figure 6A), ERβ mRNA expression (Figure 6B), and GPR30 mRNA expression (Figure 6C) were analogous in this study. We found that these parameters were not affected by age, UCMS processes, and CSS administration and were comparable among groups (*p*>0.05; Figures 6A–C).

### Effects of CSS Administration on the ERα/ERβ mRNA Expression Ratio in the Hippocampus of the UCMS Rats

The ERα/ERβ ratio was sensitively affected by the UCMS processes, and we found that this ratio was markedly decreased in the UCMS without CSS group compared with that in the other groups (*p*<0.001), indicating that the ERα/ERβ ratio may be a potential marker of the physiological symptoms in aging rats.

The ERα/ERβ ratio was also sensitively affected by CSS administration, and it was significantly higher in the UCMS with CSS group than in the UCMS without CSS group (*p*<0.001), indicating that CSS administration significantly elevated the ERα/ERβ ratio in the hippocampus of the UCMS rats (Figure 6D). We thus found that the ERα/ERβ ratio was not affected by age (*p*>0.05).

## Discussion

Although CSS is widely used to treat perimenopausal anxiety and depression in China [17], [25], the poor understanding of its mechanisms has limited the further development of this therapeutic preparation. Furthermore, because the composition of CSS is complex, understanding the mechanisms of action of CSS is critical to the assessment of its efficacy and safety. Therefore, the present study was designed to investigate the potential mechanisms of action of CSS in the treatment of perimenopausal psychological syndromes.

In the present study, we could interestingly observe both depression-like behavior and anxiety-like behavior in the UCMS rats. This UCMS model therefore resembles psychological syndromes, including depression and anxiety. In order to confirm the perimenopausal state of the aging rats (ACON and UMCS rats), we evaluated their estrous cycle by microscopic examination of vaginal smears. Interestingly, we found that the duration of estrous cycle in the CON rats was regular and short (in 4 days), whereas that in the aging rats was disturbed and longer (almost over 7 days, Figure 2B). We thus validated that these aging rats imitated perimenopausal women. In our preliminary experiments, we found that the performances in all the behavioral tests were affected by the estrous cycle (data not shown); therefore, we eliminated the rats on the estrous stage of the cycle from subsequent behavioral assessments.

The most important finding in the present study was the profound increase in the hippocampal ERα/ERβ mRNA ratio following CSS administration. Although the role of cerebral ER in anxiety and depression is controversial, this observation may be relevant to the effects of CSS. Several studies report the crucial role of ER in perimenopausal psychological symptoms [26], [27]. Ryan (2012) reported a relationship between ERα and severe depressive symptoms in women, although the role of ERβ was not confirmed [28]. Suzuki (2012) reported limited efficacy of ERβ agonists in the maintenance of functional dorsal raphe (DR) neurons to treat postmenopausal psychological problems [29]. In the present study, although ERα, ERβ, and GPR30 mRNAs were highly expressed in the hippocampus, CSS administration exerted no effects in the UCMS rats (UCMS with CSS group vs. UCMS without CSS group, Figures 6A–C). In fact, rather than showing the association of ERα, ERβ, and GPR30 expression with perimenopausal anxiety and depression, our data suggest that the ERα/ERβ ratio plays a more important role in influencing perimenopausal psychological symptoms. Several studies have demonstrated the crucial effects of the ERα/ERβ ratio on oxidative stress in patients with breast cancer [30], [31], on food intake regulation, on eating behavior [32], and on metabolic regulation [33], [34]. To our knowledge, this is the first study to report that CSS mediated an increase in the ERα/ERβ ratio (Figure 6D.), which was significantly associated with alleviation of the abnormal mood state in the UCMS rats. Therefore, the hippocampal ERα/ERβ ratio may be potentially employed as an index to evaluate the mood state in animals. In addition, investigation of the increase in this ratio as a therapeutic target for perimenopausal psychological symptoms can be considered in the future. Although the mechanisms linking the hippocampal ERα/ERβ ratio with perimenopausal psychological symptoms remain unknown, numerous reports have linked oxidative stress to the pathogenesis of anxiety and depression [35], [36], [37]. Our future bench study is to confirm the relationship between oxidative stress, the ERα/ERβ ratio, and perimenopausal psychological symptoms in animals.

Estrogen reportedly exerts a neuroprotective effect, and decreased systemic estrogen levels have been thought to play a predominant role in hormonal changes occurring during menopause, which manifest as several perimenopausal symptoms, including anxiety and depression [2], [38], [39]. Moreover, these symptoms can be improved by hormone replacement therapy [40], [41], [42]. Interestingly, the present data indicated significantly decreased hippocampal and serum E_2_ levels in aging rats (CON group vs. aging groups; Figure 5); however, they failed to show any effects of stress or CSS administration. In addition, the UCMS model failed to affect hippocampal and serum E_2_ levels; therefore, it remained unaltered by subsequent CSS administration. One possible explanation is that we found the standard deviation (SD) of the values (both serum and hippocampal) to be quite large (SD not shown in the figures). Increased fluctuations in the E_2_ levels of the animals during our experiments indicated that they are not appropriate to be used as an index in this study.

Furthermore, rigorous multicenter clinical trials are required to verify the efficacy and safety of CSS. In fact, similar to other alternative therapies, poor experimental designs have decreased the power of clinical studies on CSS [43], [44]. The findings of this study provide several clues for future clinical studies. We are now planning to conduct clinical studies to investigate the ERα/ERβ ratio in both the serum and cerebrospinal fluid (CSF) of patients suffering from perimenopausal psychological symptoms. If the ERα/ERβ ratio in serum and/or CSF shows the same variation trend as that in hippocampus, this ratio can be considered as a potential clinical index for evaluating perimenopausal psychological symptoms because both serum and CSF can be easily collected in a clinical setting.

In conclusion, our data suggest that enhancement of the hippocampal ERα/ERβ ratio plays an important role in CSS mechanisms. Furthermore, the ERα/ERβ ratio may have the potential to function as a diagnostic index and therapeutic target for perimenopausal psychological syndromes in the future.
